# Parametric Study on Low-Velocity Impact (LVI) Damage and Compression after Impact (CAI) Strength of Composite Laminates

**DOI:** 10.3390/polym14235200

**Published:** 2022-11-29

**Authors:** Shuangxi Guo, Xueqin Li, Tianwei Liu, Guangyu Bu, Jiangbo Bai

**Affiliations:** 1 AVIC Manufacturing Technology Institute, Beijing 101300, China; 2 School of Transportation Science and Engineering, Beihang University, Beijing 100191, China; 3 Jingdezhen Research Institute of Beihang University, Jingdezhen 333000, China

**Keywords:** low-velocity impact, compression after impact, composite, delamination

## Abstract

A full-scale model for predicting low-velocity impact (LVI) damage and compression after impact (CAI) strength was established based on a subroutine of the material constitutive relationship and the cohesive elements. The dynamic responses of the laminate under impact load and damage propagation under a compressive load were presented. The influences of impact energy and ply thickness on the impact damage and the CAI strength were predicted. The predicted results were compared with the experimental ones. It is shown that the predicted value of the CAI strength is in good agreement with the experimental result. As the impact energy reaches a certain value, the CAI strength no longer decreases with the increase in the impact energy. Decreasing the ply thickness can effectively improve the damage resistance and CAI strength.

## 1. Introduction

One of the advantages of composite laminates is that different fiber-reinforced materials and design angles can be selected according to the load requirements of various structures, which provides greater freedom for the structural design and ply scheme optimization. However, the obvious anisotropy and low inter-laminar strength characteristics of the composites can also lead to multiple failure modes of structures, including fiber breakage, resin cracking [1,2] and delamination [3,4,5].

The impact damage and residual strength of composite laminates have attracted lots of attention. A lot of related works have been published, and experimental testing is one of the most direct and effective research methods. Using an experimental method, Sergii [6] investigated the impact and compression after impact (CAI) behavior of thin carbon fiber epoxy plates with two different discontinuous fiber lengths. Compared with the behavior of the continuous fiber composite laminate, the results show that longer fibers provide better damage tolerance. Gliszczynski [7] presented the global failure modes of channel section profiles subjected to CAI during experimental investigations, and the laminates with different angle ply arrangements and different impact angles were discussed. Elamin [8] studied the CAI behavior of carbon-fiber foam-core sandwich composites in low temperature conditions, and the experimental tests were conducted across temperature ranging from −70 to 23 °C. The results showed that low temperature lead to a significant reduction in the CAI strength by 60%. Abdulhamid [9] experimentally studied the CAI values of tapered composite laminate with a thickness from 4 to 6 mm. It was found that the compressive behavior of the specimens is mainly governed by the discontinuity of the neutral axis in the tapered region. Wang [10] analyzed the effect of stacking sequences on delamination morphology and the compression damage propagation mode of thin laminates. The CAI tests were performed with a non-standard device to suppress the global buckling of the plates. It was concluded that the position of the initially propagating delamination is located on one side or both sides of the 0° layer. Liu [11] investigated the CAI behavior of composite panels with foam-filled hat stiffener. The experimental results show that material compression damage is caused by impact damage, and the impact location has an obvious influence on the CAI strength. Zhang [12] compared the response of tubular composite structures with/without a honeycomb core under low-velocity impact (LVI) and CAI, and the results illustrate that the core material has a greater energy absorption and a higher CAI strength.

With the development and improvement of the finite element method, numerical simulation has become more and more popular. Tian [13] proposed an equivalent damage model to predict the CAI strength of composite laminates and stiffened panels, and the numerical model achieved a high computational efficiency by utilizing the soft inclusion method. Shah [14] studied the damage tolerance and CAI values of resin-infused thermoplastic three-dimensional (3D) woven composites which were compared with the conventional resin-infused thermoset composite, and the results show that thermoplastic matrix has a better damage tolerance. Combining the experimental tests and the numerical simulation, Tuo [15] investigated the damage and failure mechanism of thin composite laminates under the CAI. Zhang [16] analyzed the influence of the off-axis angles on the LVI and CAI damage mechanism of three-dimensional woven carbon/epoxy composites, and the results indicate that the off-axis samples exhibited higher damage volumes and out-of-plane deformation. Applying the finite element method, Bull [17] studied the effect of the parameters observed from the previous studies on the CAI. It was found that toughness plays a more significant role than permanent out-of-plane deformation does, and they evidenced and quantified the role of the undamaged cone. Lu [18] numerically investigated the effect of geometries on the CAI behavior of carbon reinforced composites. The results show that width-to-thickness ratio has a significant effect on the failure load, whereas the effect of plate aspect ratio has a smaller effect. To effectively predicting the CAI strength of large-scale composite structures, Johannes [19] developed a model using stacked sub-laminates and the explicit dynamics approach. It was found that the CAI strength in [45/0/90/−45]_4s_ sub-laminate scaled laminates can be predicted accurately, and the maximum error is 10%. Yang [20] analyzed the effect of the impactor diameter on the impact damage and CAI strength, and it was found that the CAI strength increased by approximately 34~53% with the decrease in the impactor diameter.

To improve the impact resistance of the composites, researchers have developed ultra-thin prepregs in recent years, which can delay the initiation and propagation of inter-laminar and intra-laminar damage, such as delamination, matrix cracking and fiber breakage [21,22]. Cameron [23] studied the effect of using thin plies to increase the bearing strength of composite laminates, and it was found that shifting from a 100% conventional ply laminate to a 100% thin ply laminate gives an increase of 47% in the strength at the initiation of the damage. Using different formulations of thin ply composites, Cugnoni [24] evaluated the effect of the fiber and matrix constituents on the initiation of the damage and their strength. The results show that the delamination threshold load of thin laminates was increased by 15% compared to that of the conventional laminates. Saito [25] compared the impact resistance of carbon fiber reinforced polymer laminates with two different ply thicknesses, and the results show that the impact resistance of thin ply laminates is 23% higher than that of the laminates with a conventional ply thickness. However, by investigating the LVI resistance of thin ply in comparison with conventional aluminium-carbon laminates, Drodziel [26] stated a different conclusion, which was that the thin ply carbon fiber reinforced polymer neither increases the LVI resistance nor significantly changes the low impact response.

In order to evaluate the impact damage resistance and residual strength of composite materials, it is necessary to use numerical methods to simulate the progressive damage and predict the bearing capacity of the composite structure that is damaged and to study the influence of material parameters on its impact response. In this study, a Finite Element Model (FEM) for predicting the impact damage and the CAI strength of the composite laminates was established. The whole analysis process of impact damage and CAI strength was conducted using the FEM. The stress state of each element was controlled through a subroutine to simulate the initiation and propagation of in-plane damage and delamination between the plies. The impact damage and the CAI strength of the carbon fiber composite laminates were simulated, and the influence of the impact energy and ply thickness on the impact damage area and CAI strength were studied.

## 2. Experimental Tests

Standard specimens were prepared by CCF800/AC531 unidirectional reinforced prepreg with 0.14 mm thickness, and the basic mechanical properties are presented in Table 1. Where *E*_11_ and *E*_22_ are the modulus in the fiber and transverse direction, respectively, *G*_12_ is the in-plane shear modulus, *v*_12_ is Poisson’s ratio, *X_t_*, *X_c_*, *Y_t_* and *Y_c_* are the tensile strength in the fiber direction, the compressive strength in the fiber direction, the tensile and compressive strength in the transverse direction, respectively, and *S* is the in-plane shear strength. In this study, the fiber was considered to be uniformly distributed, and the composite laminate was simplified to a uniform orthotropic material. According to the ASTM standards [27,28], the specimens were cured in the autoclave. The laminates were heated to 180 °C, and then, they were cured under the curing pressure of 0.6 MPa for 150 min. The length of specimens was 150 mm, and the width was 100 mm. Thirty-two plies constitute the layup of the thick section with a stacking sequence of [45/0/−45/90]_4s_.

**Figure 1:**
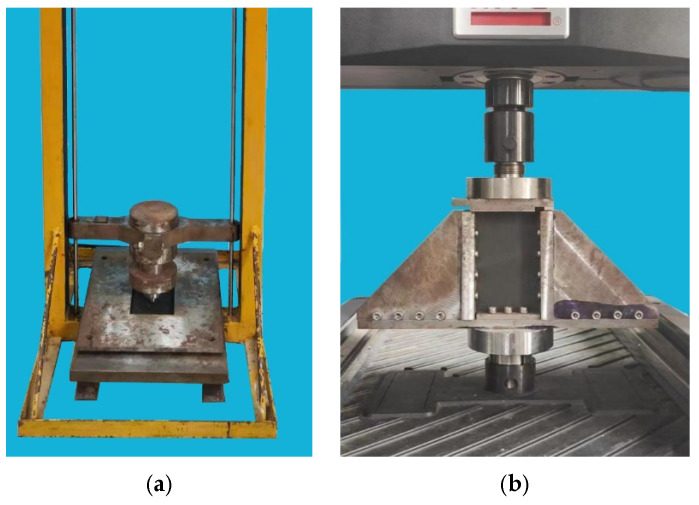
Devices for the CAI test. (**a**) Impact test device. (**b**) Compression test device.

Impact tests were performed using a drop weight testing device. The impactor weight was 5.5 kg, and it has a hemispherical shape puncher with a 16 mm diameter. Impact energy was 6.67 J/mm according to the ASTM D7136 standard, and impact energy of the specimens was 30 J in this paper. During the impact process, the specimens were calmed by a fixture with rectangular opening of 125 × 75 mm^2^. Figure 1a,b shows the impact test device and compression test device, respectively.

**Figure 2:**
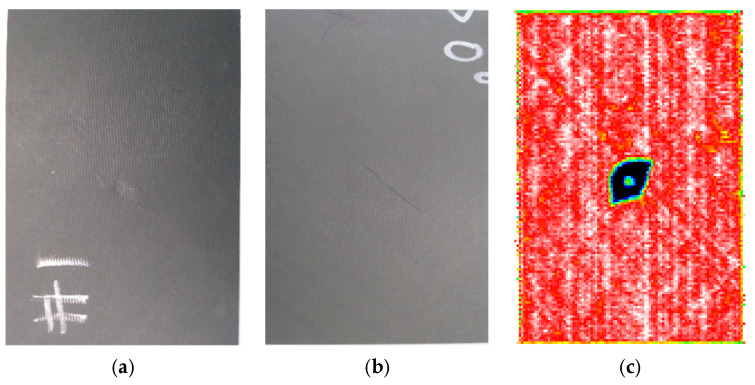
Damage of specimens after impact. (**a**) Upper surface. (**b**) Lower surface. (**c**) Image of C-scan.

**Figure 3:**
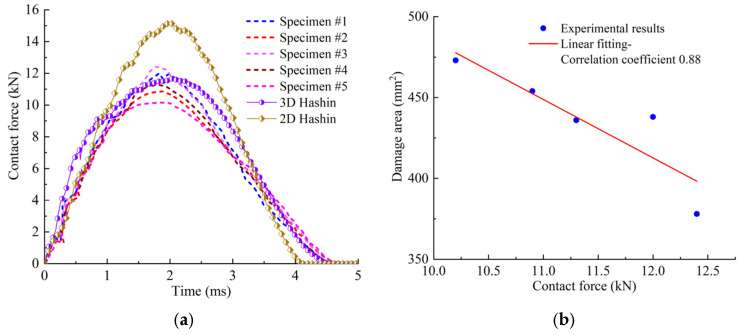
Contact forces and damage areas of specimens. (**a**) Comparison of the contact force during impact. (**b**) Relationship between damage area and contact force.

Figure 2 illustrates the damage of specimens after impact. From Figure 2, it can be seen that a pit appeared on the upper surface of the laminate, and fiber breakage and matrix cracking damage appeared on the lower surface. The shape of the pit on the upper surface was approximately circular, the matrix cracking was along the fiber direction (45° direction) on the lower surface. The delamination, matrix cracking and fiber breakage damage caused by impact in the thickness direction were obtained using a C-scan technique. The damage area obtained from the C-scan is shown in Figure 2c. The damage shape was approximately elliptical, and the damage ranges were different for the different specimens. The contact forces of 5 specimens during impact are illustrated in Figure 3a, and the maximum contact force varies from 10 to 12 kN, and it can be seen that the numerical predict curve with the 3D criterion agrees well with experimental ones, and the result with 2D criterion is much larger. The damage areas and CAI values of the specimens are listed in Table 2. It can be seen that the impact-induced damage area is negatively correlated with the maximum contact force. The relationship between the damage area and the contact force is listed in Figure 3b, and the correlation coefficient is about −0.88. It should be pointed out that there is dispersion among the different samples, but the average properties of the material are basically stable, and we verified the numerical model by comparing them with the experimental average values.

**Figure 4:**
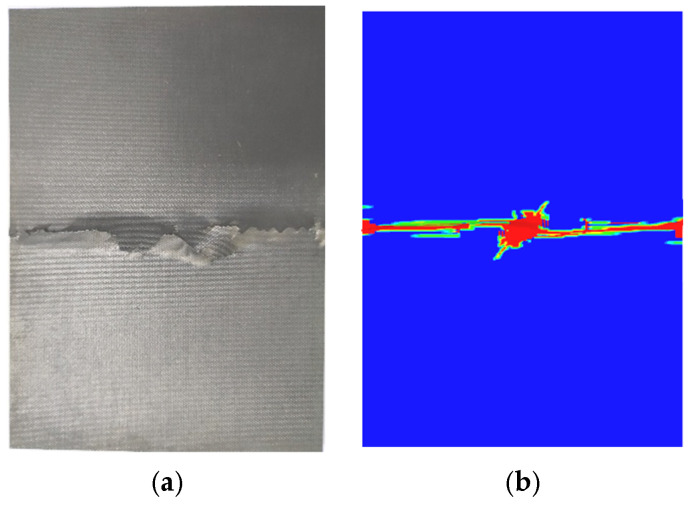
Images of specimens after compression failure. (**a**) Experimental compression failure. (**b**) Predicted compression failure.

**Figure 5:**
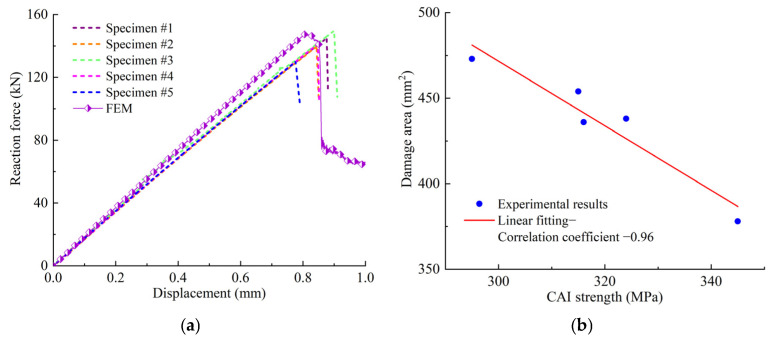
Reaction forces and CAI strengths of specimens. (**a**) Reaction forces versus displacement curves. (**b**) Relationship between damage area and CAI strength.

The impact tests were followed by the CAI tests, and the CAI device was set up according to the ASTM D7137 standard. Supports in the length and width directions of the specimens were provided on both sides to prevent bend failure due to the bending deformation, and the compressive displacement rate was 1.0 mm/min. Figure 4 shows the comparison of the typical specimens after compression failure between the experimental test and the numerical simulation. It clear that the failure modes between the experimental test and the numerical simulation are in good agreement. The failure mode is a lateral failure, and the failure area runs through the width, and the failure location appears to be in the middle of the specimen’s length. This failure mode is one of the common acceptable failure modes of the CAI, as mentioned in the ASTM D7137 standard. Figure 5a illustrates the compressive displacement versus the load curves, and the six curves are well correlated. Table 2 lists the failure compressive displacements and the CAI values of the specimens. It can be concluded that the impact damage area is negatively correlated with the CAI strength, and the relationship between the damage area and the contact force is listed in Figure 3b; the correlation coefficient is about −0.96. Additionally, the maximum difference of the CAI strengths is about 50 MPa for the five specimens.

## 3. Numerical Model

### 3.1. Failure Criterion

#### 3.1.1. Intra-Laminar Damage Model

There are a lot of investigations on the failure criteria of composite laminates. Many researchers have proposed different failure criteria, which can be divided into two types: a single mode failure criterion and a multi-mode failure criterion. The multi-mode criterion considers the contribution of different types of loads to structural failure. In this study, the 3D Hashin criterion [29] was used, and the composite damages in this criterion were classified into four types: a fiber tensile failure, a fiber compression failure, a transverse tensile failure and a transverse compression failure. The equations are:

(1) $${\left(\frac{\sigma_{11}}{X_{c}}\right)}^{2}+{\left(\frac{\tau_{12}}{S_{12}}\right)}^{2}+{\left(\frac{\tau_{13}}{S_{13}}\right)}^{2}\geq 1\;\;\;\;\sigma_{11}\geq 0$$

(2) $$\begin{array}{cc} {\left(\frac{\sigma_{11}}{X_{c}}\right)}^{2}\geq 1 & \sigma_{11}<0 \end{array}$$

(3) $$\begin{array}{cc} {\left(\frac{\sigma_{22}\;+\;\sigma_{33}}{Y_{t}}\right)}^{2}+\frac{\left(\tau_{23}^{2}\;-\;\sigma_{22}\sigma_{33}\right)}{S_{23}^{2}}+{\left(\frac{\tau_{12}}{S_{12}}\right)}^{2}+{\left(\frac{\tau_{13}}{S_{13}}\right)}^{2}\geq 1 & \left(\sigma_{22}+\sigma_{33}\right) \end{array}\geq 0$$

(4) $$\begin{array}{cc} {\left(\frac{\sigma_{22}\;+\;\sigma_{33}}{2S_{12}}\right)}^{2}+\frac{\sigma_{22}\;+\;\sigma_{33}}{Y_{c}}\left({\left(\frac{Y_{c}}{2S_{12}}\right)}^{2}-1\right)+\frac{\left(\tau_{23}^{2}\;-\;\sigma_{22}\sigma_{33}\right)}{S_{23}^{2}}+{\left(\frac{\tau_{12}}{S_{12}}\right)}^{2}+{\left(\frac{\tau_{13}}{S_{13}}\right)}^{2}\geq 1 & \left(\sigma_{22}+\sigma_{33}\right)<0 \end{array}$$

where $\sigma_{ii}$ (*i* = 1,2,3) are the normal stresses in the *i* direction, $\tau_{ij}$ (*I,j* = 1,2,3) are the shear stresses in the *ij* plane, *X_t_*, *X_c_*, *Y_t_* and *Y_c_* are the tensile strength in the fiber direction, the compressive strength in the fiber direction and the tensile and compressive strength in the transverse direction, respectively, and *S_ij_* is the in-plane shear strength. The mechanical properties of the CCF800/AC531 composite ply are listed in Table 1.

When a certain failure mode appears, the corresponding material properties should be degraded, and there will be no other failure modes. The material properties after degradation can be expressed by state variables, and the Camanho model [30] was used to characterize the material state when an in-plane failure occurred.

#### 3.1.2. Inter-Laminar Damage Model

The interface between two adjacent plies was simulated by the cohesive elements. When the interlaminar stresses exceed the strength, the failure process of the composite material is characterized by the continuous reduction of the material stiffness. The exponential constitutive model and the bilinear constitutive model are the two most popular damage constitutive models for interface damage [31,32]. The bilinear constitutive model was used in this paper, and the linear elastic stress versus strain relationship of the interface without delamination can be written as:

(5) $$\left[\begin{array}{c} \sigma_{n}\\ \tau_{s}\\ \tau_{t} \end{array}\right]=\left[\begin{array}{ccc} K_{n} & 0 & 0\\ 0 & K_{s} & 0\\ 0 & 0 & K_{t} \end{array}\right]\left[\begin{array}{c} \delta_{n}\\ \delta_{s}\\ \delta_{t} \end{array}\right]$$

where $\sigma_{n}$, $\tau_{s}$ and $\tau_{t}$ are the interlaminar normal stress and the interlaminar shear stresses, respectively, $K_{n}$, $K_{s}$ and $K_{t}$ are the corresponding stiffness, and $\delta_{n}$, $\delta_{s}$ and $\delta_{t}$ are the displacements in three directions. The delamination initiation and propagation of the interface are determined by the second nominal stress criterion and BK criterion [33], as follows:

(6) $${\left(\frac{\langle \sigma_{n}\rangle }{T}\right)}^{2}+{\left(\frac{\tau_{s}}{S}\right)}^{2}+{\left(\frac{\tau_{t}}{S}\right)}^{2}=1$$

(7) $$G^{C}=G_{n}^{C}+(G_{s}^{C}-G_{n}^{C}){\left(\frac{G_{s}^{C}+G_{t}^{C}}{G_{s}^{C}+G_{n}^{C}}\right)}^{\eta }$$

where *T* and *S* are the normal tensile strength and shear strength, respectively, $G^{C}$ is the energy release rate for mixed delamination propagation, $G_{n}^{C}$, $G_{s}^{C}$ and $G_{t}^{C}$ are the energy release rate in the normal and shear directions, and *η* is the parameter related to interface property, which was selected to be 1.0 in this paper. The property parameters of interface are listed in Table 3.

### 3.2. Finite Element Model

**Figure 6:**
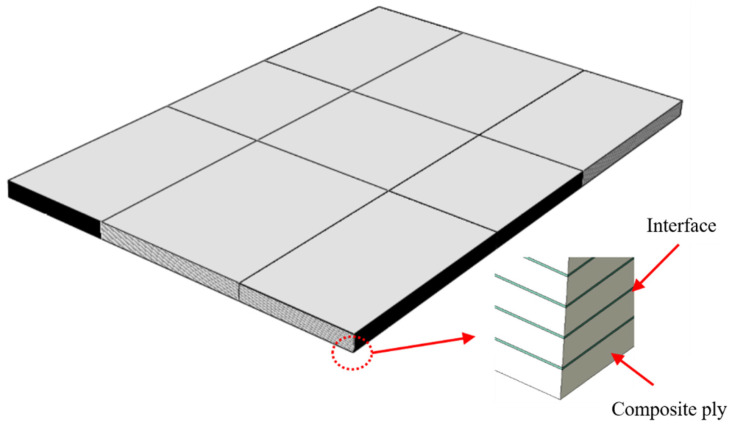
FEM of the composite laminate.

Composite laminates are made by bonding multiple fiber-reinforced composite plies. The lay-up of the laminate was [45/0/−45/90]_4s_, and the ply thickness was 0.14 mm. According to the size of composite laminate specimen specified in the test standard, an FEM of the composite laminates was established. Since the stiffness of the impactor is much greater than that of the composite laminate, the deformation of the impactor can be ignored, and the impactor was set as the rigid body in the FEM. The FEM of the composite laminate is shown in Figure 6. The composite plies were modeled by solid elements, and the interfaces were modeled by cohesive elements. Since the angles of the adjacent plies of laminates are not the same in this paper, a total of 31 interface plies were utilized. The interface plies were numbered from 1 to 31 from the impact surface to the back surface.

## 4. Impact Damage and Compressive Strength

### 4.1. Impact-Induced Damage

**Figure 7:**
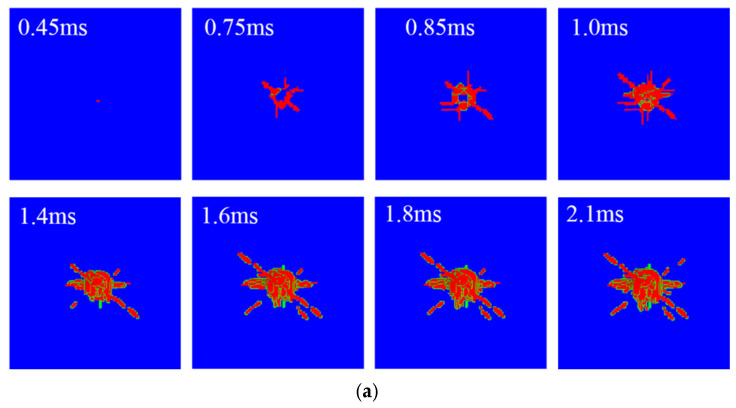

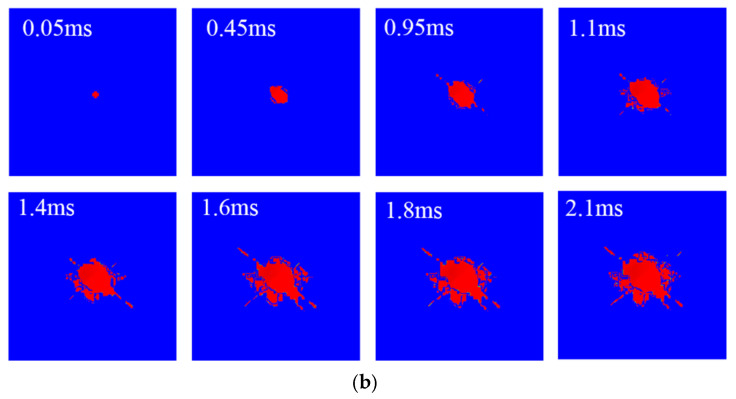
Intra-laminar failure of the laminate. (**a**) Fiber failure zone (red zone). (**b**) Matrix failure zone (red zone).

An impact damage prediction model of the composite laminate was established to calculate the response under the impact load. According to the ASTM D 7136 standard, the kinetic energy of the impactor was implemented using the initial velocity, and the impact energy was set at 30 J. Figure 7 shows intra-laminar fiber failure and the matrix cracking states at different impact times. It can be seen that matrix cracking appeared earlier than it did in the fiber due to its low strength, and the final damage zone of the fiber and matrix caused by the impact is roughly the same, which is approximately circular.

**Figure 8:**
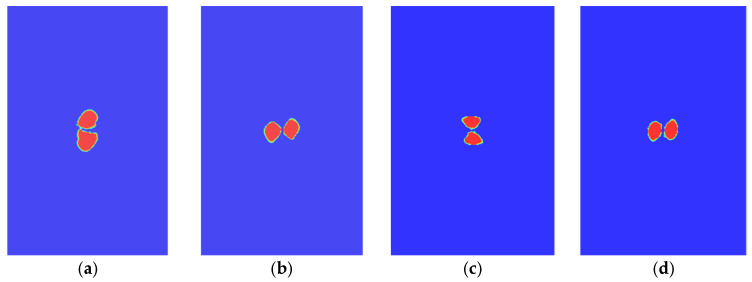
Delamination of typical interfaces. (**a**) The 15th interface. (**b**) The 18th interface. (**c**) The 20th interface. (**d**) The 26th interface.

The damage states of typical interfaces are shown in Figure 8. It is shown that the delamination shapes of these interfaces are relatively close, which are similar to the number “8”. The propagation directions of the delamination are not the same, and the delamination tends to propagate along the direction that is perpendicular to the fiber. Figure 3 shows the time history of contact force between the impactor and the laminate during the impact process. The calculated values are in good agreement with the experimental ones, which validates the established FEM.

### 4.2. Simulation of Compression Failure

The laminate state at the end of impact was used as the initial condition of the compression simulation. The failure state of the fiber, matrix and delamination at the beginning of compression was defined by field variables. Considering the storage space and computing efficiency of the computer, it was impossible to simulate the compression loading rate under real experimental conditions. The loading rate needs to be increased to ensure that the calculation time is within an acceptable range, and the enforced displacement loading rate used in this research was 50 mm/s.

**Figure 9:**
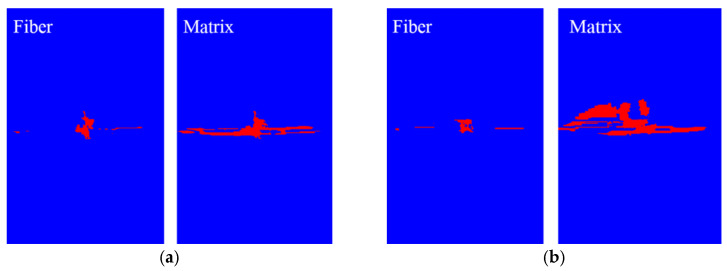
Intra-laminar failure propagation. (**a**) Intra-laminar failure of the 7th ply. (**b**) Intra-laminar failure of the 18th ply.

Figure 9 illustrate comparison between the fiber failure propagation and matrix failure propagation of typical plies. It can be seen that the failure range of fiber and matrix has a certain propagation under the compression load. The matrix damage area is much larger than the fiber damage area is due to the lower matrix strength compared with the fiber strength.

**Figure 10:**
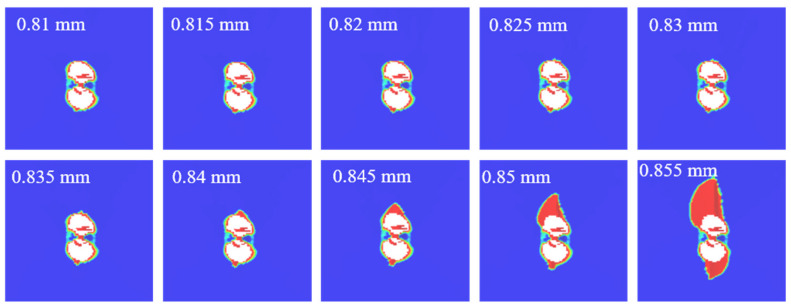
Delamination propagation with different compressive displacements.

Figure 5 compares the reaction forces predicted by the numerical model and the experiments, and the predicted curves are in agreement with experimental ones. From Figure 5, it shows that the reaction force is linearly proportional to the compression displacement in the initial stage. The reaction force begins to gradually decrease when the compression displacement reaches about 0.81 mm, which indicates that the damage began to propagate in a large area. When the displacement reaches 0.85 mm, the reaction force drops sharply, and the whole structure fails. The result indicates that when the compression displacement is less than 0.81 mm, the damage does not propagate significantly. While the compression displacement increases from 0.81 mm to 0.85 mm, the damage rapidly propagates and the structure fails, as shown in Figure 10. Table 2 shows the comparison between the CAI prediction values and the experimental average values. It illustrates that the predicted results are in good agreement with the experimental ones. The error between the predicted value using the 3D criterion and the experiment is about 4.7% for the CAI strength, which validates the presented model.

### 4.3. Influences of Damage Criterion

The selection of the damage criterion affects the prediction of the in-plane properties of the composite plies, and then, it affects the prediction of structural failure process and final damage state. The influences of the damage criteria on the prediction results of the impact damage and the CAI strength were studied through the FEM that was established in this paper. The 3D Hashin criterion is shown in Section 3.1.1, and the 2D Hashin criterion can be obtained by setting the parameters related to three directions (i.e., the thickness direction) in Equations (1)–(4) to 0. The damage characteristics and CAI strengths using the 3D and 2D Hashin criteria were calculated and compared.

**Figure 11:**
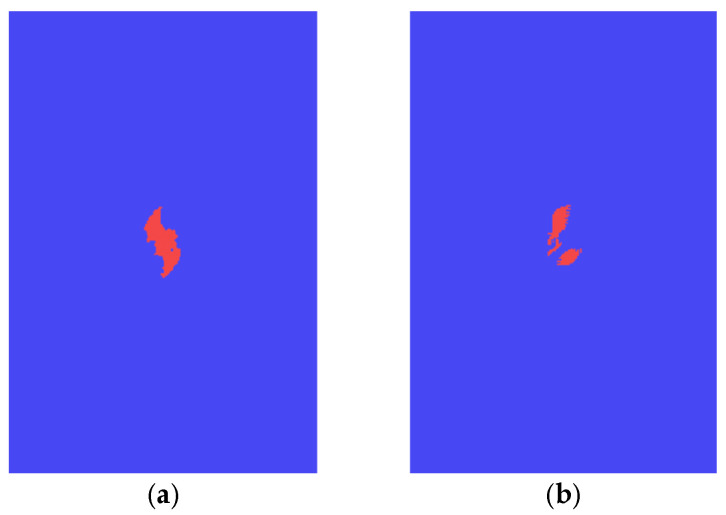
Comparison of the matrix damage. (**a**) Damage with 3D Hashin criterion. (**b**) Damage with 2D Hashin criterion.

**Figure 12:**
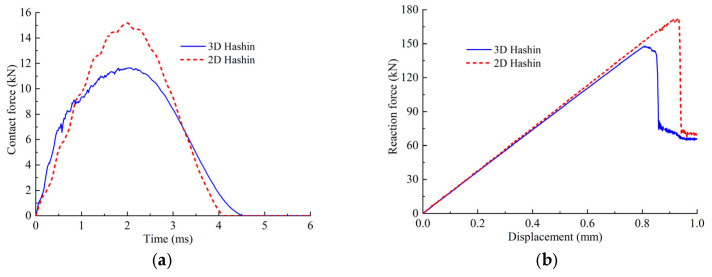
Results with two damage criteria. (**a**) The contact force. (**b**) The reaction force.

**Figure 13:**
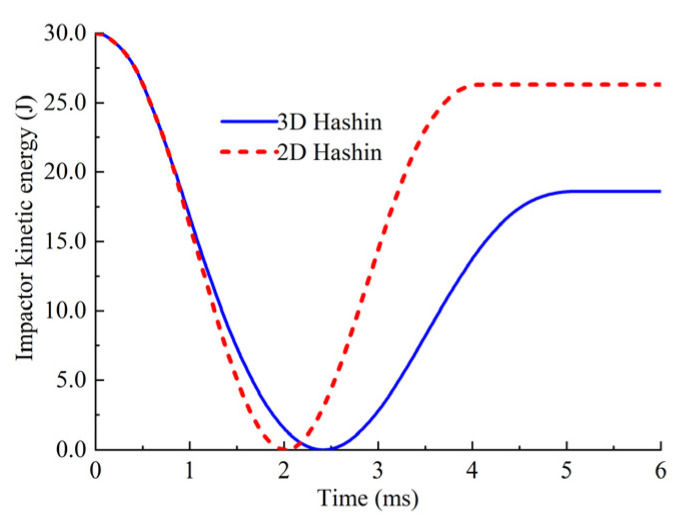
Kinetic energy variation of the impactor.

Figure 11 shows the comparison of the matrix cracking obtained by using two in-plane failure criteria with an impact energy of 30 J. It can be seen that the matrix cracking area predicted using the 3D criterion is larger than that for the 2D criterion, and the contact force versus time curves and the reaction force versus displacement curves are shown in Figure 12. The contact time between the impactor and the laminate is shorter when the 2D criterion was used, while the maximum contact force increases by about 50%. The reason for this phenomenon is that when 2D criterion was used, the energy absorbed by the laminate during the impact process is less than it was while we were using the 3D criterion, as shown in Figure 13. The reduction of the energy absorption makes the damage area smaller, thus, the remaining stiffness of the laminate is relatively larger, resulting in a short duration of the impact process and a larger impact contact force. The impact damage area obtained using the 2D criterion is relatively small, and the CAI strength is relatively larger, which illustrates that the 3D criterion is better than the 2D criterion for predicting the CAI behavior. The damage caused by out-of-plane stress should be considered in the FEM. In the following calculation, the 3D Hashin criterion was employed to simulate the composite damage of each ply.

## 5. Parameter Influences

### 5.1. Influence of Impact Energy on Compression after Impact

**Figure 14:**
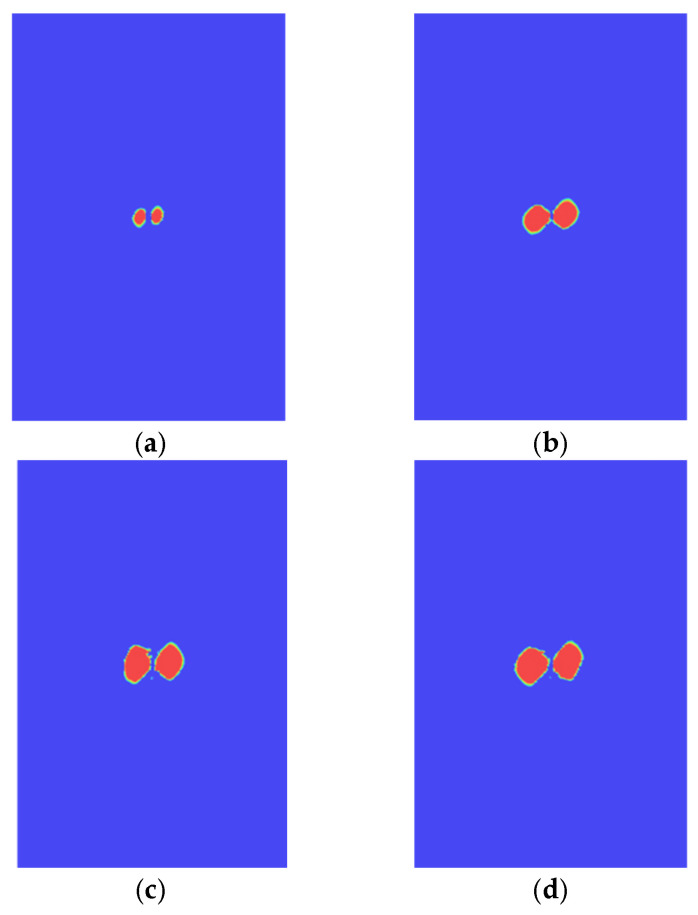
Delamination under different impact energy conditions. (**a**) Impact energy 10 J. (**b**) Impact energy 20 J. (**c**) Impact energy 40 J. (**d**) Impact energy 50 J.

The composite structures may be subjected to different impact energies, which will cause the bearing capacity of the composite structures to decline by different degrees. To study the influence of impact energy on CAI strength of the composite materials, the impact damage and CAI strengths under impact energies of 10, 20, 40 and 50 J were compared with the result of the test using 30 J. The impact damage of the composite laminate was calculated, and Figure 14 shows the comparison of delamination damage of typical interfaces caused by different impact energy conditions.

**Figure 15:**
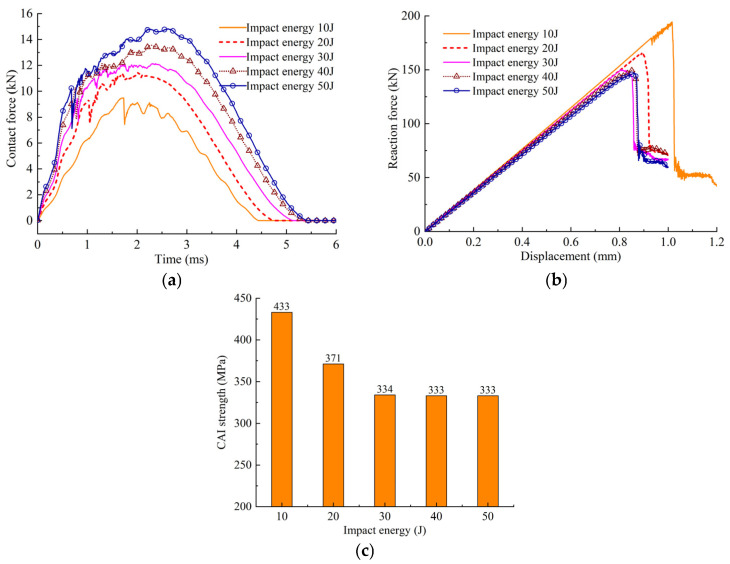
The impact and compression response under different impact energy conditions. (**a**) The contact force curves. (**b**) The compression load versus displacement curves. (**c**) CAI strength with different impact energy.

According to Figure 8 and Figure 14, it can be seen that the delamination area gradually increases when the impact energy increases from 10 to 30 J. Compared with impact energy of 30 J, increasing the impact energy to 40 and 50 J has little effect on the delamination damage area. With the increase in impact energy, the delamination damage area will not continue to increase after the impact energy exceeds 30 J. Figure 15a shows the variation of the contact force versus contact time curves under different impact energy conditions, and it can be seen that the contact force increases with the increase in the impact energy. From Figure 15b, it is clear that the reaction force gradually decreases when the impact energy increases from 10 to 30 J. After the impact energy exceeds 30 J, the maximum reaction force remains basically unchanged, illustrating that CAI strength does not decrease continually after the impact energy exceeds 30 J, as shown in Figure 15c.

**Figure 16:**
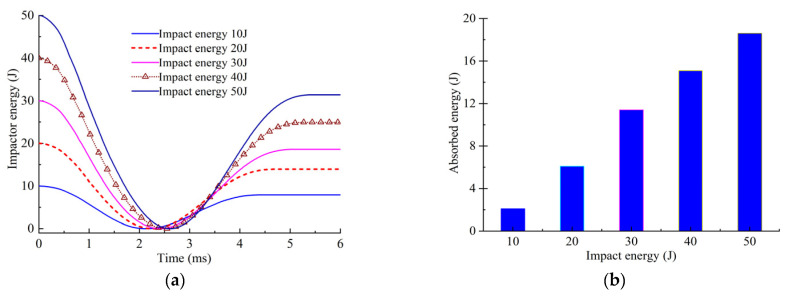
Variation of impactor energy and energy absorption. (**a**) Impactor energy curve. (**b**) Energy absorbed by composite laminate.

**Figure 17:**
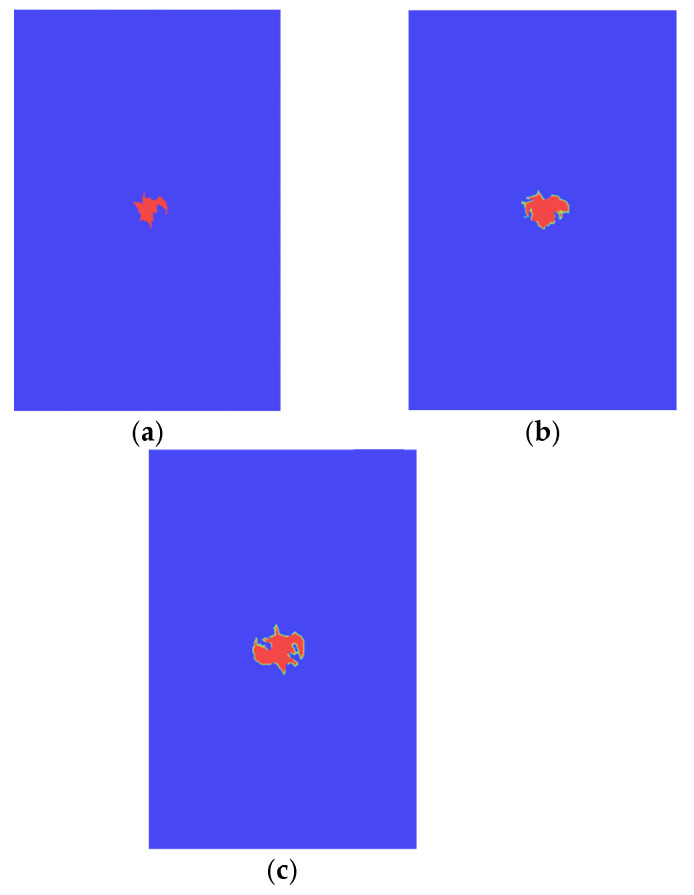
The intra-laminar failure under different impact energy conditions. (**a**) Impact energy 30 J. (**b**) Impact energy 40 J. (**c**) Impact energy 50 J.

To further explain the above phenomenon, Figure 16 shows the changes to the impactor energy and the energy absorption of the composite laminate under different impact energy conditions. It can be seen that the contact time between the impactor and the composite laminate becomes longer with the increase in the impact energy, and the energy absorption of the composite laminate also gradually increases. The in-plane damage of the typical laminates under three impact energy conditions, i.e., 30, 40 and 50 J, is shown in Figure 17, and the in-plane damage area gradually increases with the increase in the impact energy. The location of the fiber damage and matrix damage is the same as that in the delamination test, which is near the impact point. The results show that when the impact energy exceeds 30 J, the in-plane damage area of some of the plies gradually increases, which means that more impactor energy is absorbed. However, the in-plane damage and the inter-laminar delamination projection area of all of the plies do not increase significantly, indicating that the effect of impact energy on the CAI strength is not obvious after it exceeds 30 J.

### 5.2. Influence of Ply Thickness on Compression after Impact

Raman [34] shows that reducing the ply thickness may reduce the damage range of the composite structure. To study the influence of the ply thickness on compression after the impact, in this section, the impact response and CAI strength of laminates with different ply thicknesses were calculated. The results were compared with the one using conventional ply thickness of 0.14 mm, and the cured ply thicknesses and corresponding layups are shown in Table 4.

**Figure 18:**
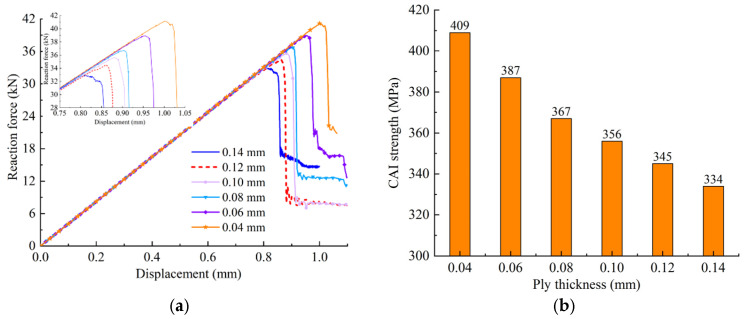
Compressive responses with different ply thicknesses. (**a**) Reaction force curve. (**b**) CAI strength with different ply thicknesses.

Figure 18 illustrates the compressive load versus displacement curves and the CAI strengths with different ply thicknesses. The reaction force versus displacement curves with different single ply thicknesses are shown in Figure 18a. It can be seen that the final reaction force increases with the decrease in single ply thickness of the composite laminate. According to Figure 18b, the CAI strength increases gradually with the decrease in the single ply thickness. Compared with the result of the 0.14 mm single ply thickness, the CAI strength increases by 75 MPa when the single ply thickness decreases to 0.04 mm, namely, the CAI strength increases by 22.4%. The results indicate that decreasing the single ply thickness can effectively improve the damage resistance and CAI strength of the composite laminates. This is consistent with Wagih [35], Huang [36] and Saito [25] who studied the effect of ply thickness on damage resistance and CAI through experiments, and they found that the CAI strength of thinner ply was 23% higher than that of a traditional ply thickness.

**Figure 19:**
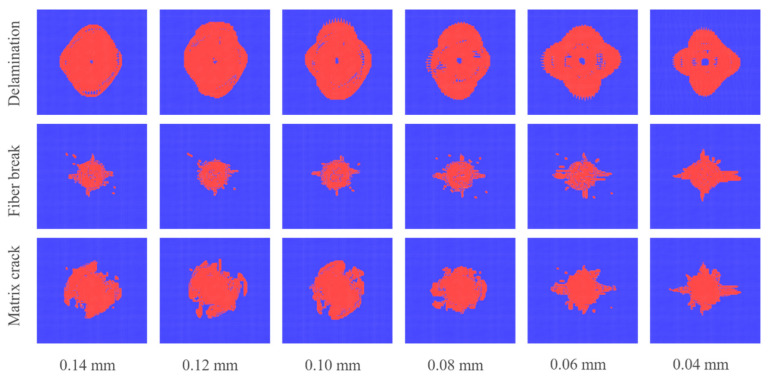
Comparison of impact damage with different single ply thicknesses.

To further analyze the mechanism of the effect of the single ply thickness on the CAI strengths, Figure 19 shows the comparison of the delamination, fiber breakage and matrix cracking areas caused by the impact. It can be seen that the single ply thickness has no obvious effect on the fiber breakage area, but the matrix cracking and delamination areas decrease with the decrease in the single ply thickness. Compared with the other conditions, the matrix cracking area with ply thicknesses of 0.06 and 0.04 mm decreases, obviously. It shows that decreasing the single ply thickness can restrain the propagation of matrix cracking caused by impact, and thus, the residual strength of the laminate in compression stage (i.e., CAI strength) is improved.

**Figure 20:**
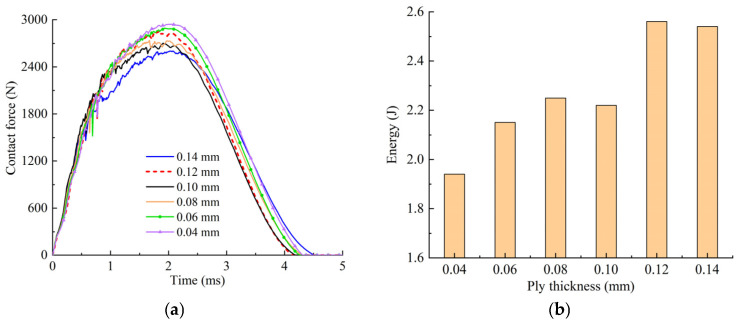
Comparison of contact forces and energy absorption with different single ply thicknesses. (**a**) Contact force curves. (**b**) Energy absorption.

The comparison of contact forces and energy absorption with different single ply thicknesses are shown in Figure 20. From Figure 20a, it can be seen that the contact force increases with the decrease in the single ply thickness, indicating that the laminate stiffness becomes larger, that is, the damage area of laminates with a thinner ply is relatively small. Figure 20b shows that the energy absorbed by the laminate decreases gradually with the decrease in the single ply thickness, which is one of the reasons for the smaller damage propagation range during the impact process.

## 6. Conclusions

An FEM for predicting the impact damage and CAI strength of composite laminates was established. The whole analysis process of the impact damage and CAI strength was conducted using the FEM. The predicted CAI strength of the composite laminate was compared with the experimental one. The influences of impact energy, damage criterion and single ply thickness on the impact damage area and CAI strength were investigated. Four important results emerging from the research are as follows:

1. The damage zone of the fiber breakage and matrix cracking caused by the impact is approximately circular.
2. The predicted CAI value is in good agreement with the experimental one, and the error is 4.7%.
3. The CAI strength decreases as impact energy increases from 10 to 30 J. After the impact energy exceeds 30 J, the CAI strength remains basically unchanged.
4. Decreasing the single ply thickness can effectively improve the damage resistance and CAI strength of the composite laminates. When the single ply thickness decreases to 0.04 mm, the CAI strength increases by 22.4%.

## Figures and Tables

**Table 1:** Properties of CCF800/AC531 composite ply.

Parameter	Value
*E*_11_/GPa	171.0
*E*_22_/GPa	8.76
*G*_12_/GPa	4.2
*v*_12_	0.25
*X_t_*/MPa	2889.0
*X_c_*/MPa	1562.0
*Y_t_*/MPa	84.1
*Y_c_*/MPa	221.0
*S*/MPa	163.0

**Table 2:** Damage areas and CAI specimens of the specimens.

Specimen	Impact Damage Area (mm^2^)	Maximum Contact Force (kN)	CAI Strength (MPa)	Failure Displacement (mm)
1	438	12.0	324	0.86
2	454	10.9	315	0.83
3	378	12.4	345	0.89
4	436	11.3	316	0.83
5	473	10.2	295	0.77
Average	436	11.4	320	0.84
Numerical	419	11.8	334	0.81
Error (%)	3.9	2.6	4.7	3.6

**Table 3:** Properties of the interface.

Parameter	Value
*G_ⅠC_*/(N/m)	329.0
*G_ⅡC_*/(N/m)	2215.0
Tensile strength/MPa	60.0
Shear strength/MPa	112.0

**Table 4:** Layups for different nominal cured ply thicknesses.

Item	Cured Ply Thickness/mm	Layup
1	0.12	[45/0/−45/90]_5s_
2	0.10	[45/0/−45/90]_6s_
3	0.08	[45/0/−45/90]_7s_
4	0.06	[45/0/−45/90]_9s_
5	0.04	[45/0/−45/90]_13s_

## Data Availability

The data presented in this study are available on request from the corresponding author.
